# Toxic Epidermal Necrolysis: A Rare Complication of Allopurinol

**DOI:** 10.7759/cureus.76848

**Published:** 2025-01-03

**Authors:** Francisco Barreto, Margarida Jardim, Carina Graça, Tiago Catanho, José J Nóbrega

**Affiliations:** 1 Internal Medicine Department, Hospital Central do Funchal, SESARAM EPERAM (SErviço de SAúde da Região Autónoma da Madeira, EPERAM), Funchal, PRT; 2 Intensive Care Department, Hospital Central do Funchal, SESARAM EPERAM (SErviço de SAúde da Região Autónoma da Madeira, EPERAM), Funchal, PRT

**Keywords:** allopurinol, allopurinol-induced ten, lyell syndrome, medical intensive care unit (micu), refractory septic shock, toxic epidermal necrolysis (ten)

## Abstract

Toxic epidermal necrolysis (TEN) is a rare, life-threatening, mucocutaneous disorder characterized by extensive epidermal detachment and necrosis, often triggered by medications. Prompt recognition and management are critical to improving outcomes.

The present article reports a case of an 80-year-old patient, with a history of gout and essential hypertension, who presented to the emergency department with pyrexia and a generalized macular rash characterized by dark centers and vesicles, accompanied by severe pruritus. The patient had recommenced allopurinol therapy one week prior, following an acute gout episode. Clinical examination revealed an extensively distributed, highly painful dark macular rash with cutaneous desquamation and a positive Nikolsky's sign, vesiculation, plasmorrhexis, and mucositis. Laboratory findings indicated elevated inflammatory markers. The patient was placed in isolation due to suspected TEN and managed with paraffin gauze dressings, temperature regulation, and fluid resuscitation. The condition was further complicated by refractory septic shock secondary to nosocomial pneumonia, ultimately resulting in the patient's demise.

This case underscores the importance of early recognition, discontinuation of the causative agent, and comprehensive supportive care in the management of TEN. The case highlights the need for a multidisciplinary approach and the role of adjunctive therapies in improving outcomes. Further studies are warranted to optimize treatment protocols for this devastating condition.

## Introduction

Toxic epidermal necrolysis (TEN) is a severe and potentially life-threatening dermatological condition, initially described by the Scottish dermatologist Alan Lyell in 1956 in the British Journal of Dermatology [1]. It is characterized by extensive epidermal detachment and mucosal erosion [2]. It is considered the most severe manifestation within the spectrum of adverse cutaneous drug reactions, with Stevens-Johnson Syndrome (SJS) representing a less extensive variant [2].

TEN typically affects more than 30% of the total body surface area and is predominantly triggered by medications such as antibiotics, anticonvulsants, and others, although infections and malignancies have also been implicated as causative factors [3].

The pathogenesis of TEN involves a complex immunological cascade, mediated by granulysin and Fas ligand interactions, leading to extensive keratinocyte apoptosis and subsequent widespread epidermal necrosis [4]. Clinical manifestations include pyrexia, cutaneous hyperalgesia, and the rapid development of widespread erythematous or purpuric macules that progress to extensive, confluent blisters and epidermal sloughing [5].

Management of TEN necessitates immediate cessation of the offending agent, comprehensive supportive care in a specialized burn unit or intensive care setting, and vigilant monitoring for complications such as fluid and electrolyte imbalances, sepsis, and multiorgan failure [6].

Despite advancements in supportive care modalities, mortality rates associated with TEN remain substantial, underscoring the critical importance of early recognition and prompt intervention in optimizing patient outcomes.

## Case presentation

An 80-year-old female patient with a medical history of gout, giant cell arteritis, hypertension, and paroxysmal supraventricular tachycardia presented to the emergency department. The patient was on a medication regimen including allopurinol, bisoprolol, and prednisolone. She reported a one-week history of asthenia, anorexia, myalgia, and dyspnea, followed by the onset of fever, generalized macular rash with dark centers, and blisters associated with intense pruritus. The previous week, she had visited the emergency department for gouty arthritis of the right hallux with superficial ulceration, after which she had resumed allopurinol intake.

**Figure 1:**
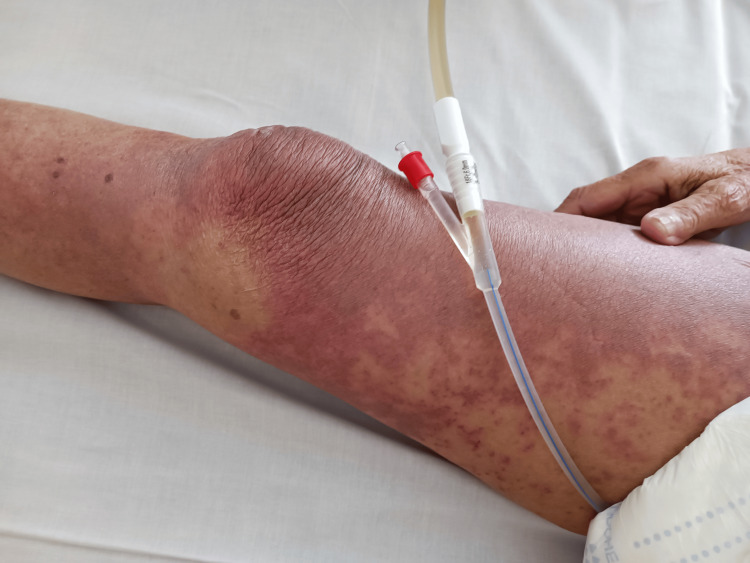
Diffuse dusky erythema with macules over the right leg and knee

Physical examination revealed a generalized dark macular rash, extremely painful to touch (Figure 1). Laboratory analysis showed leucopenia with neutropenia and lymphopenia (Table 1). The patient was admitted in isolation and received supportive treatment, wound care with paraffin gauze, and management of body temperature and fluid balance.

**Table 1:** Blood test results aPTT: Partially activated thromboplastin time; CRP: C-reactive protein; PT: Prothrombin time

Parameters		Value	Reference range
Hemogram	Hemoglobin (g/dl)	9.8	12-15 g/dl
	Leucocytes/ mm^3^	2800	3.5-10.5 (10³/uL)
	Lymphocytes/ mm^3^	700	1.2-3.4 (10³/uL)
	Platelets/mm^3^	113000	150-400 (10³/uL)
Biochemistry	Glucose (mg/dl)	274	70-100 mg/dl
	Creatinine (mg/dl)	1.94	0.6-1.2 mg/dl
	Urea (mg/dl)	83	10-40 mg/dl
	CRP (mg/dl)	193.73	<5 mg/dl
Coagulation	aPTT (sec)	32	24-35 sec
	PT (sec)	17.2	14.8- 16 sec

The patient's condition rapidly deteriorated with septic shock due to nosocomial pneumonia with methicillin-resistant Staphylococcus aureus (MRSA) and Stenotrophomonas maltophilia bacteremia associated with central venous catheter infection, with the need for immediate transfer to the intensive care unit (ICU) for advanced medical intervention. Given the severity of the patient's condition and the suspicion of a severe systemic infection, broad-spectrum antibiotics were promptly administered intravenously. The patient received targeted therapy with vancomycin and levofloxacin. The existing central venous catheter, which was considered a potential source of infection, was carefully removed and replaced under sterile conditions to ensure optimal medication delivery and minimize the risk of further complications. Due to the excruciating pain mentioned by the patient, there was a crucial need for intubation and initiation of mechanical ventilation to support breathing in order to optimize analgesia with opioids such as fentanyl. Concurrently, the medical team initiated a comprehensive treatment plan to address the underlying cause of the patient's decline.

**Figure 2:**
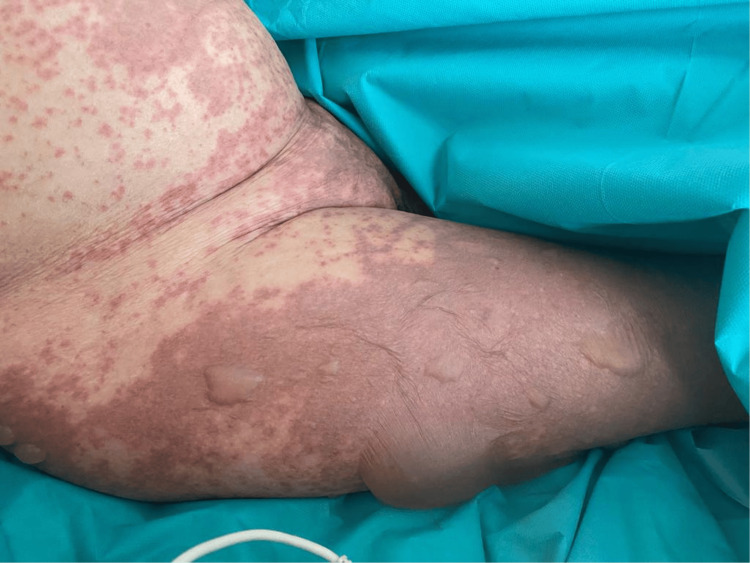
Diffuse tender erythema with macules over the abdomen and legs with flaccid bullae over the leg

**Figure 3:**
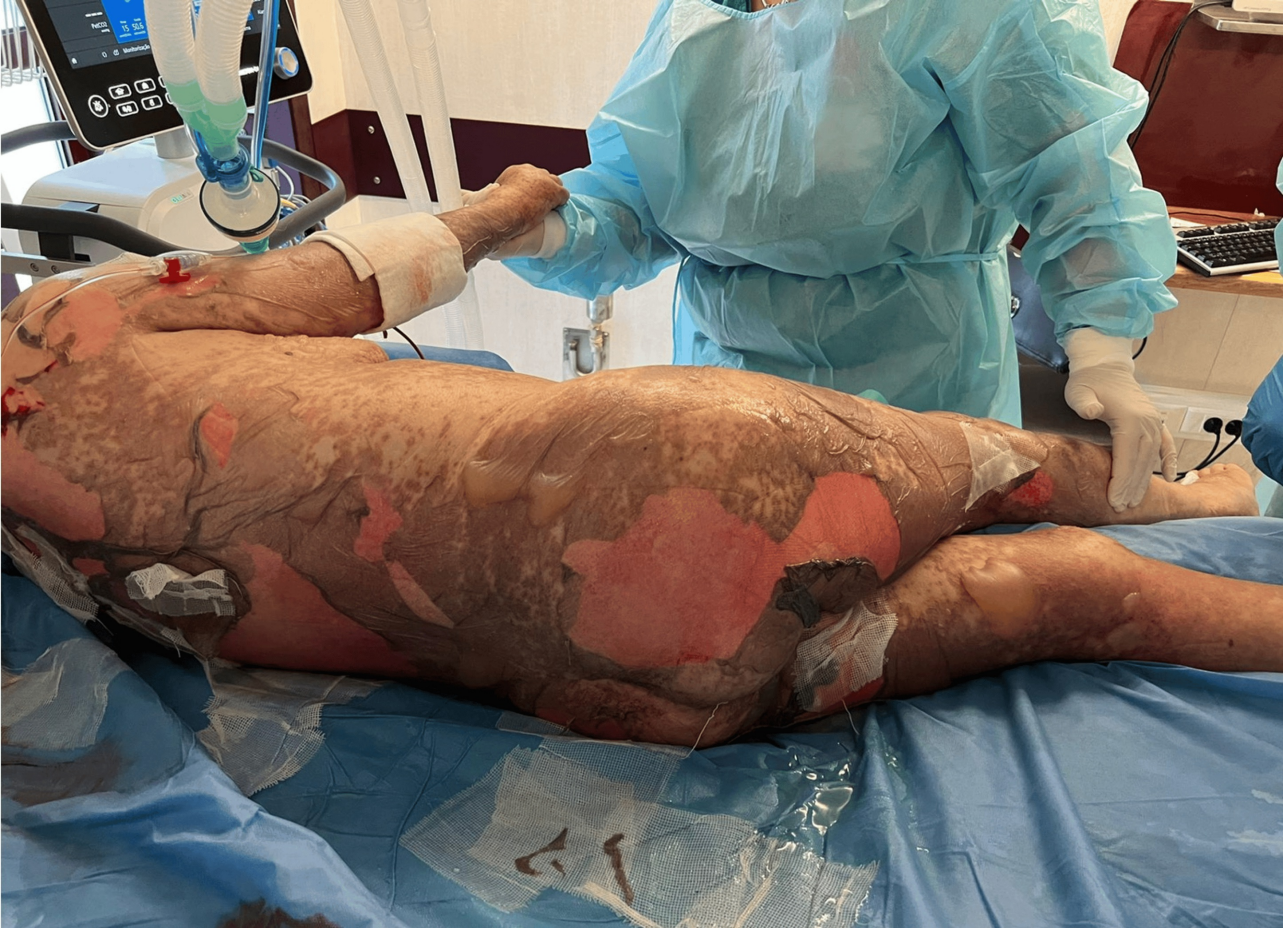
Erythema and epidermal detachment (Nikolsky's sign) on the torso, posterior side of the right leg, and in the buttock

**Figure 4:**
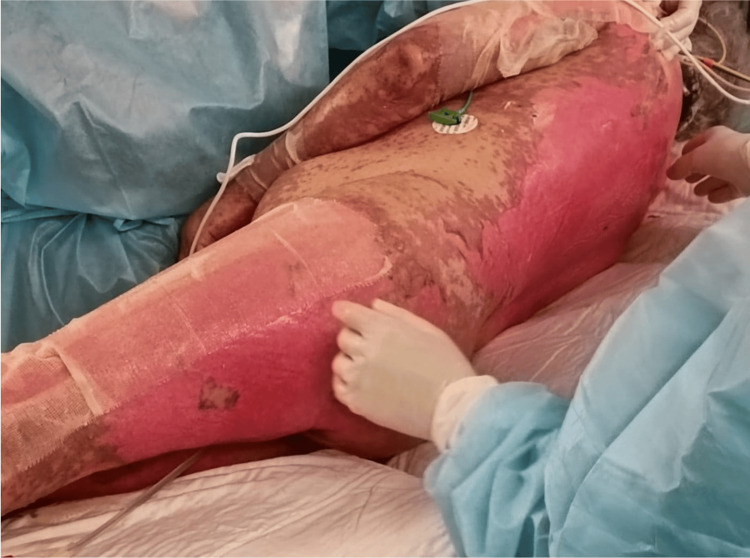
Skin detachment over the back of the patient The affected total body surface area was estimated at 80% with skin detachment.

Despite these aggressive interventions, the patient's skin lesions continued to progress at an alarming rate. The medical team observed a rapid expansion of the affected areas, with the lesions spreading to cover over 90% of the patient's body surface area within a short period (Figures 2-4), with skin detachment in almost every area affected (Nikolsky's sign).

The extensive involvement of the skin, coupled with the characteristic appearance of the lesions, led the healthcare providers to suspect a severe cutaneous adverse reaction. After a thorough clinical evaluation and consideration of the patient's history and presentation, the medical team arrived at a diagnosis of toxic epidermal necrolysis (TEN) to allopurinol.

Despite aggressive antimicrobial therapy and maximal hemodynamic support, including vasopressors, the patient's condition continued to deteriorate. Multiple organ dysfunction syndrome (MODS) eventually developed, and the patient succumbed to refractory septic shock.

## Discussion

The present case report serves as a salient exemplification of the severe and potentially life-threatening complications that can arise from TEN. This rare but critical dermatological condition, characterized by extensive epidermal detachment, can precipitate a cascade of secondary pathways that significantly complicate patient management and outcomes. TEN is typically precipitated by an adverse reaction to pharmacological agents, such as sulfonamides, allopurinol, pyrazolones, barbiturates, and antiepileptics, although in some instances, the precise etiology remains unidentified [7]. The condition's rapid progression and extensive cutaneous involvement render it a medical emergency, necessitating specialized supportive care ideally in an intensive care unit [8]. The average reported mortality rate of TEN is 25-35%, and it can be even higher in elderly patients and those with a large surface area of epidermal detachment [7].

Allopurinol is a cost-effective and widely used treatment for hyperuricemia, often resulting in good patient adherence [9]. However, its side effects can range from mild to severe, with rare but serious cases (approximately 2%) involving severe cutaneous adverse reactions such as toxic epidermal necrolysis (TEN) [9]. Monitoring and evaluating patients during allopurinol treatment is crucial. Additionally, understanding the risk factors for allopurinol-induced TEN, such as the timing of treatment initiation, genetic predisposition, drug concentration and starting dose, renal impairment, and concurrent use of diuretics, is essential.

In the present case, the patient succumbed to septic shock secondary to an infection caused by Pseudomonas aeruginosa. The development of secondary infections and organ dysfunction underscores the multisystem impact of TEN and the susceptibility of affected individuals to further medical complications. The extensive loss of skin barrier function not only predisposes patients to infections by opportunistic pathogens but also disrupts normal physiological processes, leading to fluid and electrolyte imbalances, thermoregulatory dysfunction, and increased metabolic demands [10]. These factors collectively contribute to the elevated risk of multiorgan failure observed in severe TEN cases [8,9]. Implementing strict infection control measures, including isolation precautions and meticulous hand hygiene, is crucial in minimizing the risk of nosocomial infections in these vulnerable patients [10,11].

The SCORTEN (Severity-of-Illness Score for Toxic Epidermal Necrolysis) is a validated assessment tool used to estimate the likelihood of in-hospital death for patients with TEN [12]. This prognostic score incorporates seven independent factors, with each factor contributing one point to the total, with a higher score indicating a greater probability of mortality. In this case, the patient's SCORTEN calculation yielded six points (Table 2), which corresponds to a predicted mortality rate superior to 90%.

**Table 2:** SCORTEN score SCORTEN: Severity-of-Illness Score for Toxic Epidermal Necrolysis, TEN: Toxic epidermal necrolysis, BSA: Body surface area

Factor	Points
Age > 40 years	1
Heart rate > 120/min	1
Underlying malignancy	1
Skin detachment >10% of BSA on day one	1
Serum urea > 28mg/dL (10 mmol/L)	1
Serum bicarbonate < 20 mEq/L (20 mmol/L)	1
Serum glucose > 250 mg/dL (14 mmol/L)	1

The multifaceted treatment approach employed in this case reflects the complex nature of TEN management. Supportive care forms the cornerstone of treatment, encompassing meticulous wound care, fluid and electrolyte balance maintenance, temperature regulation, and nutritional support [10,11,13]. These measures are crucial in maintaining physiological stability and promoting skin healing. Wound care in TEN patients is particularly challenging, often requiring specialized dressings and techniques to protect the denuded skin while facilitating re-epithelialization [13]. Fluid management must be carefully balanced to prevent both dehydration and fluid overload, as TEN patients can lose significant amounts of fluid through their damaged skin [10,11].

Concurrently, the implementation of targeted antimicrobial therapy highlights the critical need to address and prevent infections in patients with compromised skin integrity. This approach requires a delicate balance between providing necessary antimicrobial coverage and avoiding the overuse of antibiotics, which can lead to the development of resistant organisms [10]. The selection of antimicrobial agents must consider both the patient's clinical presentation and local patterns of antimicrobial resistance [11].

This dual approach of supportive and targeted interventions seems crucial to manage TEN cases effectively. Moreover, continuous monitoring and adjustment of treatment strategies are also important while the patient's condition evolves. The intensive care required for TEN patients often extends beyond the acute phase, with prolonged hospital stays and potential long-term complications necessitating ongoing medical attention and rehabilitation.

## Conclusions

In conclusion, this complex case of TEN serves as a powerful reminder of the challenges inherent in managing severe dermatological emergencies. It emphasizes the need for rapid diagnosis, comprehensive supportive care, vigilant infection control, and a multidisciplinary approach to patient management. The case also highlights the ongoing need for research and innovation in the field of severe cutaneous adverse reactions, with the ultimate goal of improving patient outcomes and reducing the burden of this devastating condition.

The extensive skin involvement and mucosal lesions described are characteristic of severe cutaneous adverse reactions such as Stevens-Johnson syndrome or toxic epidermal necrolysis. The patient's compromised immune status, evidenced by the leucopenia and subsequent infections, likely contributed to the severity of her condition. The development of nosocomial infections and septic shock underscores the critical importance of infection prevention measures and early, aggressive management in such cases.
